# The Modification of Cell Wall Properties Is Involved in the Growth Inhibition of Rice Coleoptiles Induced by Lead Stress

**DOI:** 10.3390/life13020471

**Published:** 2023-02-08

**Authors:** Kazuyuki Wakabayashi, Kouichi Soga, Takayuki Hoson, Harue Masuda

**Affiliations:** 1 Department of Biology, Graduate School of Science, Osaka Metropolitan University, Sumiyoshi-ku, Osaka 558-8585, Japan; 2 Department of Biological Sciences, Graduate School of Science, Osaka City University, Sumiyoshi-ku, Osaka 558-8585, Japan; 3 Urban Resilience Research Center, Osaka Metropolitan University, Sumiyoshi-ku, Osaka 558-8585, Japan

**Keywords:** cell wall extensibility, cell wall polysaccharide, coleoptile, growth inhibition, lead (Pb), rice

## Abstract

Lead (Pb) is a widespread heavy metal pollutant that interferes with plant growth. In this study, we investigated the effects of Pb on the mechanical and chemical properties of cell walls and on the growth of coleoptiles of rice (*Oryza sativa* L.) seedlings grown in the air (on moistened filter paper) and underwater (submerged condition). Coleoptile growth of air-grown seedlings was reduced by 40% by the 3 mM Pb treatment, while that of water-grown ones was reduced by 50% by the 0.5 mM Pb. Although the effective concentration of Pb for growth inhibition of air-grown coleoptiles was much higher than that of water-grown ones, Pb treatment significantly decreased the mechanical extensibility of the cell wall in air- and water-grown coleoptiles, when it inhibited their growth. Among the chemical components of coleoptile cell walls, the amounts of cell wall polysaccharides per unit fresh weight and unit length of coleoptile, which represent the thickness of the cell wall, were significantly increased in response to the Pb treatment (3 mM and 0.5 mM Pb for air- and water-grown seedlings, respectively), while the levels of cell wall-bound diferulic acids (DFAs) and ferulic acids (FAs) slightly decreased. These results indicate that Pb treatment increased the thickness of the cell wall but not the phenolic acid-mediated cross-linking structures within the cell wall in air- and water-grown coleoptiles. The Pb-induced cell wall thickening probably causes the mechanical stiffening of the cell wall and thus decreases cell wall extensibility. Such modifications of cell wall properties may be associated with the inhibition of coleoptile growth. The results of this study provide a new finding that Pb-induced cell wall remodeling contributes to the regulation of plant growth under Pb stress conditions via the modification of the mechanical property of the cell wall.

## 1. Introduction

Lead (Pb) is one of the most abundant heavy metal contaminants in both terrestrial and aquatic environments. Pb is not biodegradable and accumulates in organisms. Pb has no biological function, but it is highly toxic to living organisms even at low concentrations and causes disease, such as neurotoxicity and kidney damage in animals [1]. In plants, a prominent Pb toxicity is the inhibition of organ growth [2,3,4,5]. The toxic effects of Pb on cellular functions have been extensively studied; for example, incorporated Pb stimulates the production of reactive oxygen species (ROS), elevates the levels of lipid hydroperoxide, and increases the activities of antioxidant enzymes, while it decreases the chlorophyll contents and thus declines the photosynthetic activity [2,3,4,5,6,7,8,9,10]. Alterations in metabolic and biochemical processes may cause physiological changes in plant development under Pb stress conditions. In addition to metabolic and biochemical alterations, it has been shown that Pb disturbed the microtubule organization in meristem cells and interfered with cell division in roots [11,12]. Therefore, the suppression of cell division in root meristem may be associated with the inhibition of root growth under the Pb stress condition. In addition to the increment of cell number, the increase in cell volume is an important factor determining the growth rate of plant organs. Lane et al. [13] showed that Pb interfered with auxin-induced cell elongation in segments of wheat (*Triticum aestivum*) coleoptiles. They also showed that Pb treatment decreased the deformation ability of turgid coleoptile segments under constant inflection load. These results imply that Pb affected the mechanical properties of the cell walls when it inhibited auxin-induced growth of the segments. However, it has not been clarified whether Pb directly affects the cell wall mechanical properties in growing stem organs.

Plant cell walls surround each protoplast and provide protoplasts with mechanical rigidity. Furthermore, cell walls play an important role in the regulation of growth and morphogenesis in plants [14,15,16]. Cell wall extensibility, a parameter of cell wall mechanical property, represents the capacity of the cell wall to extend and thus the parameter is related to the elongation capacity of plant cells [15,16,17]. Cell walls of growing plant tissues are mainly composed of polysaccharides, such as cellulose and a variety of matrix polysaccharides. The quantities and chemical structures of cell wall polysaccharides are considered to be factors determining cell wall extensibility [14,15,17,18]. In addition to polysaccharides, the cell wall of gramineous (cereal) plants, such as rice (*Oryza sativa*), wheat, and maize (*Zea mays*), contain a significant amount of phenolic acid monomers, such as ferulic acid (FA), which are ester-bound to matrix polysaccharides [19,20]. Some FA residues undergo a coupling reaction to produce diferulic acid (DFA), which forms cross-links between matrix polysaccharides [21,22]. The formation of the cross-linkages by phenolic acids makes the cell wall mechanically rigid [20].

Plant cell walls play an efficient barrier to the entry of heavy metals into the protoplast [23]. Heavy metals, including Pb, increased the thickness of root cell walls in several plant species, such as *Vicia faba*, *Oryza sativa*, and *Allium cepa*, and protonemata cells of *Funaria hygrometrica* [24,25,26,27]. The thickening of the cell wall is associated with a decrease in cell wall extensibility [15,17,28]. An increase in cell wall thickness in seedling stems is accompanied by an increase in cell wall constituents, especially polysaccharides [17,28]. It is expected that Pb increases levels of cell wall constituents, such as polysaccharides and cell wall-bound DFA and FA in coleoptiles, which may promote the cell wall thickening and the formation of cross-linkages within the cell wall and thereby decrease cell wall extensibility. In the present study, we investigated the above possibility using air- and water-grown rice coleoptiles. The present results revealed a key role of the plant cell wall in the regulation of organ growth under heavy metal stress conditions.

## 2. Materials and Methods

### 2.1. Plant Materials and Growth Conditions

Caryopses of rice (*Oryza sativa* L. cv. Koshihikari) were sterilized in ca. 1% (*v*/*v*) sodium hypochlorite solution for 1 h and then soaked in deionized water for two days at 25 °C in the dark. Germinated caryopses were grown for four days in the dark at 25 °C under two different cultural conditions: on moistened filter paper (air-grown) and underwater (water-grown). For the cultivation in air, germinated caryopses were placed on one layer of filter paper in a cylindrical polycarbonate box (15 cm in diameter and 8 cm in height) which contained 30 mL of 2 mM MES-KOH buffer (pH 6.0) containing different concentrations (0, 0.1, 0.3, 1.0, and 3.0 mM) of PbCl_2_ (Wako Pure Chemical Industries, Ltd., Osaka, Japan). The cultivation underwater was as follows: germinated caryopses were submerged in a test solution (ca. 7 cm in depth) in a polycarbonate cylinder (4 cm in diameter and 11 cm in height). Each cylinder contained 80 mL of 2 mM MES-KOH buffer (pH 6.0) containing different concentrations (0, 0.25, 0.5, and 1.0 mM) of PbCl_2_. For the transplant experiment, water-grown seedlings that had been grown for 2 days in the dark at 25 °C in 2 mM MES-KOH buffer (pH 6.0) were immediately transferred to the same buffer containing 0 and 0.5 mM PbCl_2_ and grown for a further 2 days in the same conditions. On the days after planting, the lengths of coleoptiles and roots were measured with a commercially obtainable ruler. After the measurement, coleoptiles were excised. Since the coleoptile excised from air-grown seedlings contained a primary leaf inside, a vertical slit was made at the basal portion of the coleoptile and then the primary leaf was removed using forceps. After the removal of the primary leaf, the fresh weight of the coleoptile was measured using an electric balance. In contrast, the primary leaf inside the coleoptile scarcely grew under submerged conditions. So, the coleoptile excised from water-grown seedlings was used readily for the measurement of fresh weight. All manipulations were performed under dim green light (ca. 0.09 μmol m^−2^ s^−1^ at handling level). The growth experiment was repeated at least three times. The amounts of cell wall constituents, the cellular osmotic concentration, and the Pb content were determined using three samples obtained from three independent experiments. The measurement of the cell wall mechanical properties was repeated twice using samples obtained from two independent experiments.

### 2.2. Assay of Pb Content

Shoots consisted of coleoptile and the inner primary leaf and roots were used for the assay of Pb content. Seedlings were grown in the air for 4 days in the presence (1 mM and 3 mM) or absence of PbCl_2_, as described in the above section. After the cultivation, seedlings were washed several times with deionized water, and then shoots and roots were excised. Their fresh weights were measured using an electric balance. Shoots and roots excised from the control and 1 mM Pb-treated seedlings were put in Teflon vessels and immediately oven-dried at 60 °C for 2 days. The dried samples were digested completely with HNO_3_/HClO_4_ (2:1, *v*/*v*) solution at 140 °C for 24 h. After the acidic solution was evaporated completely, the digested samples were dissolved in 0.1 N HNO_3_ and analyzed for Pb content using inductive coupled plasma–mass spectrometry (ICP-MS) (SPQ 9700; Hitachi High-Tech Science Corp., Tokyo, Japan). For the measurement of Pb content in cytoplasmic fluid, shoots excised from 1 mM and 3 mM Pb-treated seedlings and roots from 1 mM Pb-treated ones were boiled for 10 min in 10 mL of 80% ethanol. The ethanol extract was dried in Teflon vessels. Dried samples were digested with HNO_3_/HclO_4_ and then dissolved in 0.1 N HNO_3_, as described above. The Pb content in the cytoplasmic fluid of 3 mM Pb-treated roots could not be analyzed because roots of air-grown seedlings hardly grew at this concentration.

### 2.3. Measurement of the Osmotic Concentration of Cell Sap

The extraction and collection of cell sap were carried out according to the method of Ooume et al. [29]. The coleoptiles obtained from air-grown and water-grown seedlings were put in a plastic mini-column and then immediately frozen with liquid nitrogen. The cell sap was collected from frozen–thawed coleoptiles by centrifugation at 1500× *g* for 10 min at 4 °C. The osmotic concentration of the collected cell sap was measured with a vapor pressure osmometer (Model 5500C; Wescor, Logan, UT, USA).

### 2.4. Measurement of the Mechanical Properties of the Cell Wall

The coleoptiles prepared from air-grown and water-grown seedlings were immediately boiled for 10 min in 80% ethanol and then stored in fresh 80% ethanol. Before the measurement of cell wall mechanical properties, ethanol-fixed samples were rehydrated for several hours. Cell wall extensibility was measured with a tensile tester (RTM-25; Toyo Baldwin Co., Tokyo, Japan) [30]. The subapical region (1–2 mm below the tip) of air-grown coleoptile was fixed between two clamps 2 mm apart, and stretched by lowering the bottom clamp at a speed of 20 mm/min to produce a stress of 10 g. In the case of water-grown plants, a segment 10 mm in length was excised from the tip of the coleoptile. The segment was fixed between two clamps 2 mm apart and stretched at the same speed to produce a stress of 4 g. Cell wall extensibility (μm/g) was determined by measuring the rate of the increase in stress just before it reached the maximum stress (4 g and 10 g for water-grown and air-grown coleoptiles, respectively).

### 2.5. Fractionation of Cell Wall Constituents

Cell wall materials were prepared and fractionated according to the method of Wakabayashi et al. [31]. Briefly, cell wall preparation was treated with 1 M NaOH to extract ester-linked phenolic acids. Then, the residual material was extracted with 17.5% NaOH containing 0.02% NaBH_4_. The fraction extracted with 17.5% NaOH was neutralized with acetic acid. After the extraction of cell wall-bound phenolic acids from the 1 M NaOH solution as described below, the remaining solution was combined with the 17.5% NaOH extracts, and designated as the matrix polysaccharide fraction. The alkali-insoluble fraction was designated as the cellulose fraction. The cellulose fraction was dissolved with 72% sulfuric acid. The total sugar content in each fraction was determined by the phenol-sulfuric acid method [32] and expressed as glucose equivalents.

### 2.6. Determination of Cell Wall-Bound Phenolic Acids

Analysis of cell wall-bound phenolic acids was carried out according to the method of Wakabayashi et al. [31]. Ester-linked phenolic acids liberated from the cell wall with 1 M NaOH (see above) were recovered into ethyl acetate by acidification. The liberated phenolic acids were analyzed using an HPLC system equipped with a reversed-phase column and a photodiode array detector with a gradient elution of methanol. FA and *p*-coumaric acid (*p*-CA) were identified and quantified using authentic *trans*-FA and *trans*-*p*-CA (Wako Pure Chemical Industries, Ltd., Osaka, Japan). The peaks of DFA isomers were identified and quantified using response factors [33].

### 2.7. Statistical Analysis

For each measurement, the means and the standard errors of the means (SE) were calculated. The significance of differences among the treatments with different Pb concentrations was analyzed using Tukey’s HSD test (*p* < 0.05). The significance of differences between the control and single Pb treatment was analyzed using Student’s *t*-test (*p* < 0.05). 

## 3. Results

### 3.1. Effects of Pb on Air-Grown Seedlings

**Figure 1:**
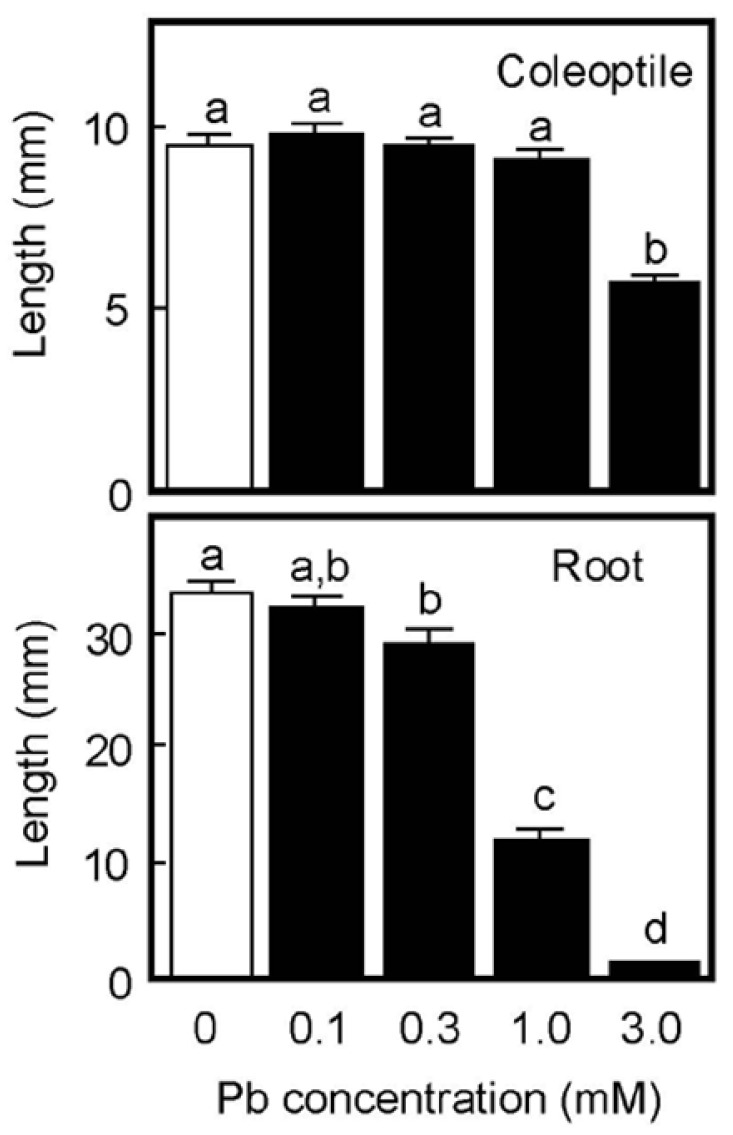
Effects of Pb on the growth of coleoptiles and roots of air-grown rice seedlings. Germinated caryopses were planted on filter paper containing a 2 mM MES-KOH buffer (pH 6.0) with or without different concentrations of Pb and then grown for 4 days in the dark. Data are means ± SE (*n* = 18–20). Different letters above the bars represent statistically significant differences (Tukey’s HSD test, *p* < 0.05).

When germinated rice caryopses were grown for 4 days on moistened filter paper, the lengths of primary roots and coleoptiles reached about 33 mm and 10 mm, respectively, in the absence of Pb (Figure 1). Root growth was significantly inhibited by the 0.3 mM Pb treatment and the inhibitory effect significantly increased in a concentration-dependent manner. Roots scarcely elongated at 3 mM Pb. In contrast, coleoptile growth was not inhibited by Pb up to 1 mM, but was significantly inhibited at 3 mM (Figure 1). Growth of the first leaf inside the coleoptile synchronized with that of the coleoptile until the start of leaf emergence. Pb also inhibited the growth of first leaves at 3 mM, but lower concentrations did not affect leaf growth; the lengths of first leaves on 4 days were 8.4 ± 0.3, 8.4 ± 0.2, 8.3 ± 0.3, 8.1 ± 0.2, and 4.8 ± 0.3 mm (each *n* = 18–20) for 0, 0.1, 0.3, 1, and 3 mM Pb, respectively. These results suggest that the inhibitory effect of Pb on the growth of aboveground organs in air-grown rice seedlings was much smaller than that of roots. 

Next, the accumulation of Pb in shoots consisting of coleoptile and first leaf and in roots of air-grown seedlings was analyzed by ICP-MS. When germinated caryopses were grown for 4 days on moistened filter paper containing a buffer solution with or without 1 mM Pb, root growth was substantially inhibited by 1 mM Pb, but shoot growth was not, as shown in Figure 1. The Pb contents in the 1 mM Pb-treated shoot and root were 119 ± 25 and 1025 ± 86 ng/organ, respectively (*n* = 3), while the contents in the control seedlings were negligible (Pb contents in the control shoot and root were 0.1 and 0.4 ng/organ, respectively). We further examined the Pb accumulation in the cytoplasmic fluid of shoots and roots that had been grown for 4 days in the presence of 1 mM and 3 mM Pb. The Pb contents in the cytoplasmic fluid of the 1 mM and 3 mM Pb-treated shoots were 0.33 ± 0.07 and 3.10 ± 0.29 μg/g fresh weight (FW), respectively, while that of the 1 mM Pb-treated roots was 4.29 ± 0.18 μg/g FW (*n* = 3, respectively). The Pb content in 3 mM Pb-treated roots could not be analyzed because roots hardly grew at this concentration (Figure 1). The calculated concentrations of Pb in the cytoplasmic fluid were 1.6, 15, and 21 μM for the 1 mM Pb-treated, the 3 mM Pb-treated shoots, and the 1 mM Pb-treated roots, respectively.

**Figure 2:**
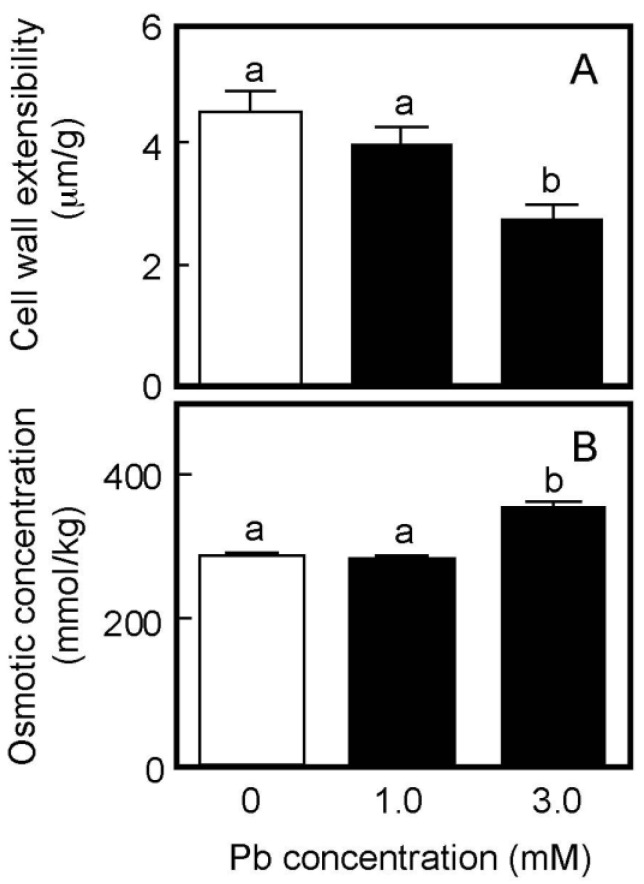
Effects of Pb on cell wall extensibility (**A**) and the cellular osmotic concentration (**B**) in coleoptiles of air-grown rice seedlings. Growth conditions are shown in Figure 1. (**A**) The cell wall extensibility of the upper region of coleoptiles was measured with a tensile tester. Data are means ± SE (*n* = 16–18). (**B**) The osmotic concentration of the cell sap obtained from coleoptiles was measured with a vapor pressure osmometer. Data are means ± SE (*n* = 3). Different letters above the bars represent statistically significant differences (Tukey’s HSD test, *p* < 0.05).

Cell wall extensibility and the osmotic concentration of air-grown coleoptiles are shown in Figure 2. Treatment with Pb at a concentration of 1 mM did not affect either cell wall extensibility or the cellular osmotic concentration (Figure 2A,B), similar to the effect on coleoptile growth. The Pb treatment at 3 mM significantly decreased cell wall extensibility and increased the cellular osmotic concentration, when it inhibited coleoptile growth (Figure 1).

**Figure 3:**
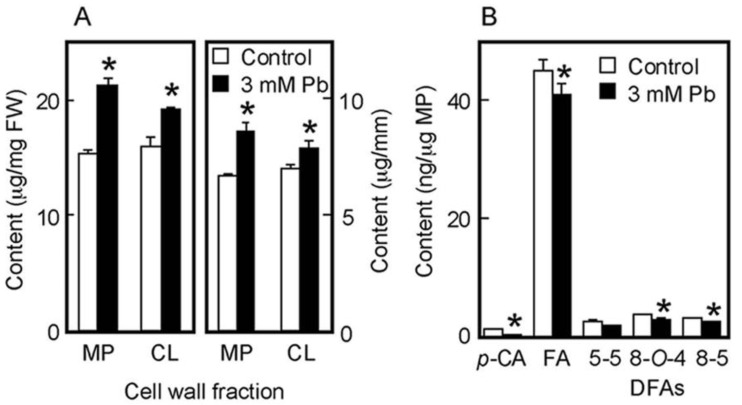
Effects of Pb on the amounts of cell wall polysaccharides (**A**) and cell wall-bound phenolic acids (**B**) in air-grown rice coleoptiles. Coleoptiles were grown for 4 days in the presence or absence of 3 mM Pb. (**A**) The sugar content in each cell wall fraction was determined by the phenol-sulfuric acid method. Amounts of cell wall polysaccharides were expressed on the basis of unit length and unit fresh weight (FW) of the coleoptile. MP, matrix polysaccharides; CL, cellulose. (**B**) Phenolic acids were analyzed by the HPLC and their amounts were expressed on the basis of unit matrix polysaccharide (MP) content. *p*-CA, *p*-coumaric acid; FA, ferulic acid; DFAs, diferulic acids. Data are means ± SE (*n* = 3). * Mean values were significantly different between the control and Pb treatment (Student’s *t*-test, *p* < 0.05).

The chemical properties of cell walls are considered to be factors determining the mechanical properties of the cell wall. We next analyzed the amounts of cell wall polysaccharides and cell wall-bound phenolic acids in air-grown coleoptiles. Cell wall polysaccharides were fractionated into two fractions, the matrix polysaccharides and cellulose. The amount of matrix polysaccharides was almost equivalent to that of cellulose in coleoptiles grown for 4 days in the air (Figure 3A). On the basis of unit length and unit fresh weight of coleoptile, Pb treatment at 3 mM significantly increased the amounts of both matrix polysaccharides and cellulose (Figure 3A).

Cell walls of gramineous plants contain phenolic acid monomers, such as FA and *p*-coumaric acid (*p*-CA). Our previous study showed that the cell walls of dark-grown rice shoots contained three predominant DFA isomers: 5-5, 8-*O*-4, and 8-5 DFA [34]. On the basis of unit matrix polysaccharide content, Pb treatment at 3 mM significantly decreased the amounts of both phenolic acid monomers, although amounts of *p*-CA were substantially lower than those of FA (Figure 3B). Furthermore, among DFA isomers, the amounts of 8-*O*-4 and 8-5 DFAs in Pb–treated coleoptiles were significantly lower than those in control ones (Figure 3B).

### 3.2. Effects of Pb on Water-Grown Seedlings

**Figure 4:**
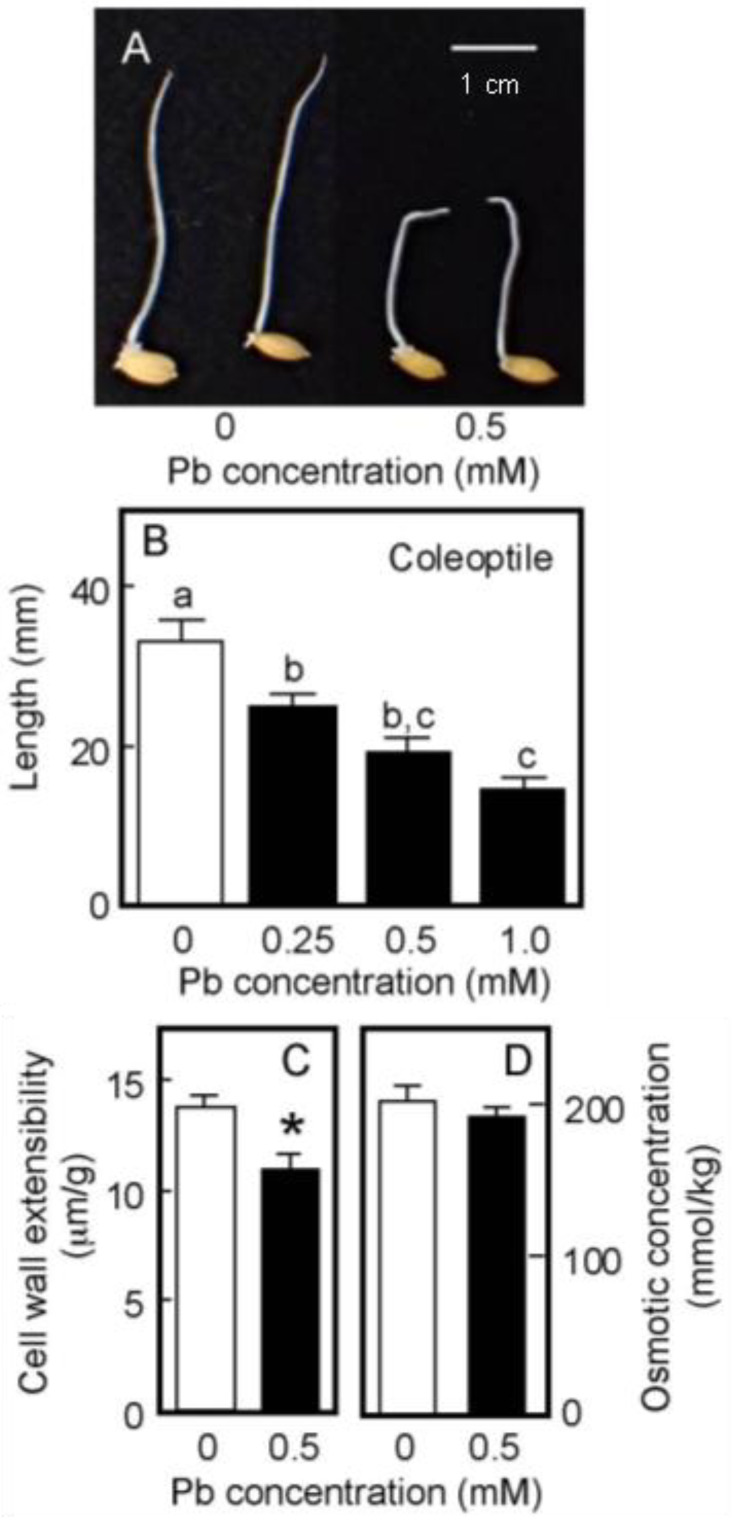
Effects of Pb on the growth of coleoptiles (**A**,**B**), the cell wall extensibility (**C**), and the cellular osmotic concentration (**D**) in coleoptiles of water-grown rice seedlings. Germinated caryopses were submerged in a 2 mM MES-KOH buffer (pH 6.0) with or without different concentrations of Pb and then grown for 4 days in the dark. (**A**) Photograph showing seedlings grown for 4 days under submerged conditions. Scale bar = 1 cm. (**B**) The length of water-grown coleoptiles. Data are means ± SE (*n* = 18). Different letters above the bars represent statistically significant differences (Tukey’s HSD test, *p* < 0.05). (**C**) The cell wall extensibility of the upper region of coleoptiles grown for 4 days with or without 0.5 mM Pb was measured. Data are means ± SE (*n* = 16–18). * Mean values were significantly different between the control and Pb treatment (Student’s *t*-test, *p* < 0.05). (**D**) The osmotic concentration of the cell sap obtained from coleoptiles that had been grown for 4 days with or without 0.5 mM Pb was measured. Data are means ± SE (*n* = 3).

Rice is a semiaquatic plant and its coleoptile grows faster underwater than in air [35,36]. In contrast, the root formation and the growth of the primary leaf inside the coleoptile are strongly suppressed when caryopses are germinated underwater [35]. As shown in Figure 4A, the root formation was strongly suppressed under submerged conditions, even in the absence of Pb. When germinated caryopses were grown for 4 days underwater, the length of the control coleoptiles reached approximately 35 mm (Figure 4B). In contrast to the air-grown seedlings, the growth of water-grown coleoptiles was significantly inhibited by the treatment with 0.25 mM Pb and the inhibitory effect increased with increasing Pb concentration. Coleoptile growth was reduced by about 50% by the 0.5 mM Pb treatment (Figure 4B).

The effects of Pb treatment at 0.5 mM on cell wall extensibility and the cellular osmotic concentration of water-grown coleoptiles are shown in Figure 4C,D. The Pb treatment significantly decreased cell wall extensibility, but did not affect the cellular osmotic concentration. Furthermore, when seedlings grown under submerged conditions for 2 days without Pb were transferred to the Pb-containing medium, Pb at 0.5 mM significantly inhibited the coleoptile growth afterward and it also significantly lowered cell wall extensibility (Table 1). 

**Figure 5:**
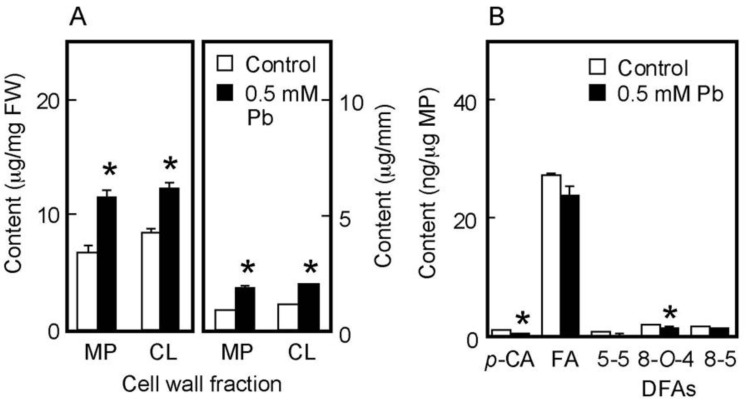
Effects of Pb on the amounts of cell wall polysaccharides (**A**) and cell wall-bound phenolic acids (**B**) in water-grown rice coleoptiles. Coleoptiles were grown under submerged conditions for 4 days in the presence or absence of 0.5 mM Pb. (**A**) The sugar content in each cell wall fraction was determined by the phenol-sulfuric acid method. Amounts of cell wall polysaccharides were expressed on the basis of unit length and unit fresh weight (FW) of the coleoptile. MP, matrix polysaccharides; CL, cellulose. (**B**) Phenolic acids were analyzed by the HPLC and their amounts were expressed on the basis of unit matrix polysaccharide (MP) content. *p*-CA, *p*-coumaric acid; FA, ferulic acid; DFAs, diferulic acids. Data are means ± SE (*n* = 3). * Mean values were significantly different between the control and Pb treatment (Student’s *t*-test, *p* < 0.05).

The amount of cell wall polysaccharides and cell wall-bound phenolic acids in water-grown coleoptiles (Figure 5A,B) were smaller than those in air-grown ones (Figure 3A,B). On the basis of unit length and unit fresh weight of the coleoptile, Pb treatment at 0.5 mM significantly increased the amounts of matrix polysaccharides and cellulose (Figure 5A). The amounts per unit matrix polysaccharide content of phenolic acid monomers and dimers were lower in Pb-treated coleoptiles than in control ones, particularly the amounts of *p*-CA and 8-*O*-4 DFA in Pb-treated coleoptiles, which were significantly lower than those in control ones (Figure 5B). The effects of Pb on the amounts of cell wall constituents were similar in air- and water-grown coleoptiles.

## 4. Discussion

Plant cell expansion is caused by the influx of water into the cell and the osmotic concentration of the cell sap provides the driving force for water uptake. In this context, an increase in osmotic concentration of the cell sap is expected to promote the growth rate, while a decrease slows it down. The cellular osmotic concentration, along with cell wall extensibility, is thought to be involved in the regulation of plant growth. In the present results, Pb had no negative effect on the osmotic concentration in air- and water-grown coleoptiles (Figure 2B and Figure 4D), suggesting that the cellular osmotic concentration is not related to the growth inhibition induced by Pb. In contrast, the Pb treatment decreased cell wall extensibility of both air- and water-grown coleoptiles, when it inhibited their growth (Figure 1, Figure 2A and Figure 4B,C, Table 1). It is suggested by these results that the decrease in the ability of the cell wall to extend is associated with the inhibition of coleoptile growth in response to the Pb exposure. The relationship between the growth inhibition of stem organs and the decrease in cell wall extensibility has been extensively examined in studies of environmental stimuli and plant hormones on stem growth [17,30,37,38,39].

The quantitative changes in cell wall constituents underlie the modification of mechanical properties of the cell wall [15,17]. The amounts of cell wall polysaccharides per unit fresh weight and per unit length of the stem show the proportion and the cross-sectional mass of the cell wall in the stem organ, respectively, and thus those values are thought to represent the thickness of the cell wall [28]. On these bases, the amounts of matrix polysaccharides and cellulose in Pb-treated coleoptiles were higher than those in control ones in both cultivation conditions (Figure 3A and Figure 5A), indicating that the cell wall thickness of Pb-treated coleoptiles was greater than that of control ones. The increase in the thickness of the cell walls results in a decrease in the cell wall extensibility of stem organs [28,30,37,40]. Therefore, Pb-induced cell wall thickening may be primarily involved in the decrease in cell wall extensibility in rice coleoptiles.

In addition to cell wall polysaccharides, the increases in the amounts of DFA and FA were associated with a decrease in the ability of the cell wall to extend in gramineous shoots [20,34,41,42]. The present results, however, showed that the levels of cell wall-bound DFAs, FA, and *p*-CA in Pb-treated coleoptiles were lower than those in control ones (Figure 3B and Figure 5B), suggesting that Pb decreased the concentration of DFA-mediated cross-linkages within cell wall architecture. These results suggest that cell wall-bound phenolic acids were not involved in the Pb-mediated decrease in cell wall extensibility. As for the effect of Pb on cell wall-bound phenolic acids, the Pb treatment only slightly affected the ratio of the total amount of three DFA isomers to the amount of FA, which was 0.20 and 0.19 for the control and Pb-treated coleoptiles grown in air and 0.15 and 0.14 for the control and Pb-treated ones grown underwater, respectively (calculated using data in Figure 3B and Figure 5B). These results suggest that Pb scarcely affects the coupling step of FA to produce DFA. Therefore, the decreases in DFA levels in Pb-treated coleoptiles may be attributed to the reduced FA level. The Pb treatment decreased the amounts of both FA and *p*-CA that are synthesized via the phenylpropanoid pathway [43]. Therefore, Pb may affect the reactions in the pathway and/or the feruoylation and coumaroylation of matrix polysaccharides, such as arabinoxylans [20,44,45].

Plant cell walls are able to bind metal cations and a large number of heavy metals incorporated into plants were localized in the cell walls [2,46,47]. Therefore, plant cell walls function not only as a barrier limiting the penetration of heavy metals but also as a sink for the accumulation of heavy metals [23]. Plant cell walls serve to sequester heavy metals from the cytoplasm, as do phytochelatins and metallothioneins, proteins that bind heavy metals. Because the cell wall can accumulate and immobilize a significant amount of heavy metals, Pb-induced cell wall thickening is thought to enhance the defense mechanism against the Pb stress [23]. The present study showed that Pb induced the cell wall thickening in coleoptiles and that the thickening caused a decrease in cell wall extensibility. Therefore, Pb-induced cell wall thickening may contribute not only to the defense strategy against Pb stress but also to the growth regulation of the aboveground organ by modifying the cell wall’s mechanical properties. 

At present, the mechanism by which Pb promotes cell wall thickening in coleoptiles is not clarified. Pb treatment stimulated the production of ROS in plant cells [7,8,48]. Although ROS have toxic effects on cellular functions, they act as signaling molecules in stress-induced cellular responses in plants. Among ROS, hydrogen peroxide is thought to be involved in the structural modification processes of the cell wall in response to abiotic stresses [49]. The application of hydrogen peroxide affected plant growth responses, such as root gravitropism [50]. Therefore, it is conceivable that hydrogen peroxide signaling may be involved in Pb-induced cell wall thickening in rice coleoptiles. In addition, Pb may affect the autolytic activity of the cell wall. Plant cell walls contain various kinds of enzymes that are involved in cell wall remodeling [51,52]. Since Pb is able to bind to acidic sugar residues of matrix polysaccharides [23,46], it is likely that Pb accumulated within the cell walls may interfere with the action and activity of enzymes involved in the degradation of cell wall polysaccharides. This possibility remains to be clarified in a future study.

**Figure 6:**
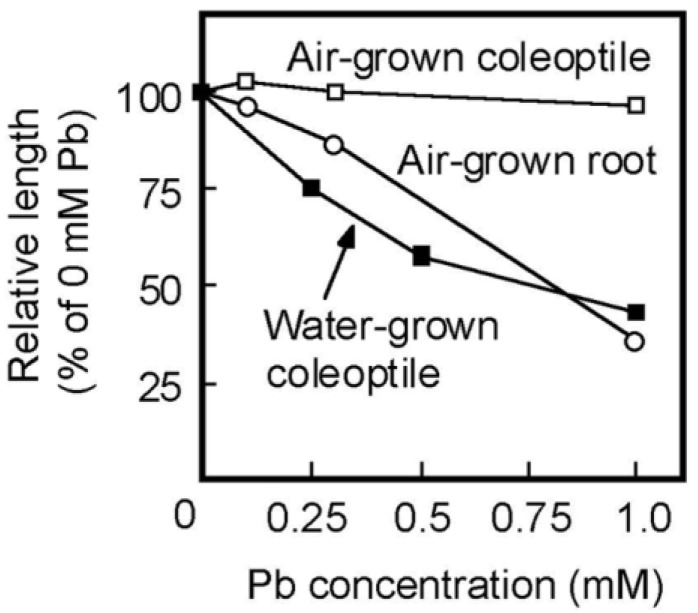
Dose–response curves of Pb on the growth of coleoptiles of air- and water-grown seedlings and of roots of air-grown seedlings. Lengths of coleoptiles and roots are shown as a percentage of the control (0 mM Pb) value. Values were calculated using the data in Figure 1 and Figure 4B. Mean values are shown.

Pb strongly inhibits root growth. So, it has been believed that the inhibitory effect of Pb on the growth of plant aboveground organs is attributed to the inhibition of root development [2]. The present results showed that Pb inhibited the coleoptile growth of water-grown seedlings, which did not develop roots (Figure 4A), suggesting that Pb directly inhibits the growth of aboveground organs. In the case of rice caryopses, the cell division ceases about 60 h after sowing and the coleoptile growth afterward is mainly due to cell elongation [36]. Therefore, the results of the transplant experiment (Table 1) suggest that Pb inhibited the cell elongation process of coleoptiles by reducing cell wall extensibility. In air-grown seedlings, the effective concentration of Pb for the inhibition of coleoptile growth was much higher than that of root growth (Figure 1). However, the dose–response of Pb for the inhibition of the growth of water-grown coleoptiles was similar to that of air-grown roots (Figure 6). Furthermore, when concentrations of Pb in the cytoplasmic fluid were elevated to the order of ten μM, severe growth inhibition was observed in shoots and roots of air-grown seedlings. It is suggested by these results that there are no apparent differences in the organ susceptibility to Pb between aboveground organs and roots in rice seedlings.

## 5. Conclusions

Pb treatment increases the thickness of the cell walls of rice coleoptiles irrespective of cultivation conditions, which may decrease cell wall extensibility. The decrease in cell wall extensibility is associated with the inhibition of coleoptile growth. It is conceivable that the growth inhibition of stem organs by other heavy metals also involves cell wall remodeling similar to that of Pb. Finally, water-grown rice seedlings may serve as a good experimental system to investigate how the cell walls in the aboveground organs of plants resist the penetration of heavy metals into the cells. 

## Figures and Tables

**Table 1:** Effects of Pb on the growth and cell wall extensibility of water-grown rice coleoptiles. Germinated caryopses were grown for 2 days in a 2 mM MES-KOH buffer (pH 6.0) (Initial), and then the seedlings were immediately transferred to the same buffer with or without 0.5 mM Pb. The transferred seedlings were grown for a further 2 days (the control and Pb treatment). The cell wall extensibility of the upper region of the coleoptile was measured. Data are means ± SE (*n* = 15–18). * Mean values were significantly different between the control and Pb treatment (Student’s *t*-test, *p* < 0.05).

Treatment	Coleoptile Length (mm)	Cell Wall Extensibility (μm/g)
Initial	14.8 ± 0.9	11.2 ± 0.4
Control	38.6 ± 2.2	14.6 ± 0.8
Pb treatment	26.8 ± 2.6 *	10.3 ± 1.1 *

## Data Availability

The data presented in this study are available upon request from the corresponding author.
